# Comparison of the Changes in Blood Glucose Level During Sedation with Midazolam and Propofol in Implant Surgery: A Prospective Randomized Clinical Trial

**Published:** 2014-09

**Authors:** Nasser Kaviani, Farzad Koosha, Mina Shahtusi

**Affiliations:** a Dept. of Anesthesiology, School of Dentistry, Isfahan University of Medical Sciences, Isfahan, Iran.; b Student, School of Dentistry, Isfahan University of Medical Sciences, Isfahan, Iran.; c Postgraduate Student of Pediatrics, School of Dentistry, Isfahan University of Medical Sciences, Isfahan, Iran.

**Keywords:** Sedation, Midazolam, Propofol, Blood glucose, Dental implant

## Abstract

**Statement of the Problem:** Reducing the patients' stress can prevent, or at least, limit the increase in blood glucose level.

**Purpose:** The study compares the effect of propofol and midazolam on blood glucose level in the patients undergoing dental implant surgery. The effect of pre-operational stress on blood glucose level during the surgery is also evaluated.

**Materials and Method:** This prospective randomized clinical trial recruited 33 patients undergoing dental implant surgery and divided into two groups. Conscious sedation was performed by midazolam in one group and with propofol in another group. The pre-operational stress was scored and the blood glucose level was measured in 4 different stages; before the operation, two minutes after the local anesthetic injection; thirty minutes after the onset of operation and at the end of the operation. The results were analyzed by employing ANOVA and Pearson test. The p Value was adopted 0.05 and the confidence coefficient was assumed 95%.

**Results:** The average levels of the blood glucose in midazolam and propofol group were 93.82 mg/dl and 94 mg/dl before the operation which displayed a meaningful increase of blood glucose level in both groups as the operation went on. The values were 103.76 mg/dl for midazolam and 108.56 mg/dl for the propofol group (*p*< 0.05) at the end of the operation.

No statistically significant difference was found in the average blood glucose level between two groups in the different stages of the operation (*p*= 0.466). The Pearson correlation coefficient test revealed a higher increase in the blood glucose level in the patients with a higher pre-operational stress score (r= 0.756, *p*< 0.001).

**Conclusion:** Based on the results yielded by this study, patients who receive venous sedation, either by midazolam or propofol, experience increase in the blood glucose level while undergoing an operation. No statistically significant difference was detected between midazolam and propofol.

## Introduction

Oral and dental operations provoke stress in the patients. In most cases, this stress activates the sympathetic system which subsequently is followed by an increase in the blood glucose level. On the contrary, in some patients, parasympathetic system is activated by this stress as a reflex phenomenon and results in hypoglycemia [1-4].

Sudden changes in the blood glucose level are generally attributable to the patient stress, operation-related stresses and the injection of anesthetics containing epinephrine; which may eventually cause problems for both patient and dentist. Therefore, it is recommended to monitor the blood glucose level constantly and try to keep it in its normal range of 72-99 mg/dl [5-8].

As pre-operational stress is one of the factors which can alter the patient's blood glucose level. It is important to benefit from the stress-control techniques such as conscious sedation to decrease the blood glucose level [9-12].

Conscious sedation can be induced by various methods. Regarding to the easy access to the medications like midazolam and propofol that have a rapid and short- lasting effect, the intravenous infusion technique has been incredibly employed, although it has been reported that propofol increases the blood lipid level [13-16].

In 2006, Oztekin et al. studied the effect of midazolam and propofol on patients undergoing coronary artery bypass graft (CABG) and concluded that patients who received both of these medications showed an increase in the blood glucose level though there were no meaningful difference between the two examined groups and no participant showed blood glucose readings beyond the critical levels [16].

In 2009, Kitamura et al. studied the effect of sevoflurane and propofol on blood glucose level and found that both groups receiving these medications showed an increase in the blood glucose level and the data was more prominent in the sevoflurane group [17]. As propofol is a fast-acting short-lasting medication, it is often recommended for intravenous sedation [17].

Considering the metabolic effects of this medication, this article aims to study the effect of propofol and midazolam on blood glucose level in patients undergoing dental implant surgery .It also evaluates the effect of pre-operational stress on the blood glucose level during this surgery. The patients undertaking the dental implant surgery are mainly the aged people who generally exhibit increased levels of blood glucose, therefore, it is crucial to control and prevent further prompting of the blood glucose level in this population.

## Materials and Method

33 patients aged between 35 and 60 years, with no disease (ASA I) were recruited for this prospective randomized clinical trial study.

The participants had to undergo dental implant surgery; 2 or 3 dental implants in the maxilla. They had no positive history of diabetes or cortisone intake. They also had no contraindication for taking midazolam and propofol. After taking a full medical history, all participants were informed about the nature of the study and asked to sign a voluntary consent form for their contribution in this study.

Thirty minutes before the operation, patients filled a dental anxiety scale questionnaire (revised type of DAS-R), a valid international form used for stress assessment [18]. The Dental anxiety scale (DAS) questionnaire was translated into Persian by the researchers and then was validated by a psychologist and a linguist to ensure that the questionnaire expresses the same meaning as the original English counterpart.

The blood glucose levels of the patients were measured on the dental unit by employing a glucometer (Accu-Chek; Active, UK). The accuracy of the glucometers of this study was assessed in Mohajery laboratory using a standard glucose solution (100 mg/dl). Glucose strips were also tested by being wet in a standard glucose solution and then tested by the glucometer.

The feasibility of these glucometers for such purposes has been approved in previous published studies [19-20].

The patients were randomly assigned for the propofol or midazolam group using the table of random numbers. The patients were catheterized on the hand by an Angiocath 20 gauge catheter (Mediplus, India) and were monitored by pulse-oxymeter. This medical device shows the respiratory rate, heart rate, saturation and the blood pressure simultaneously.

The injection of 1mg of midazolam (Tehran Chemie Pharmaceutical co.; Iran) was performed in the midazolam group and it was repeated every 2 minutes, when needed, until the conscious sedation condition was started.

In the propofol group, 10 mg of lidocaine was injected initially and was followed by administration of 25µg/kg/min propofol (Dongkook pharma Co; Korea). After 5 minutes, local anesthetic agent containing lidocaine and epinephrine was injected by infiltration technique, restricted to a maximum of 3 carpules.

Aspiration was done to ensure prevention of injection in to the veins. The blood glucose level was measured 2 minutes after the local anesthetic injection.

To achieve a complete anesthesia, the surgery began after 30 minutes and both groups had their blood glucose level measured another time using the aforementioned technique. Eventually, the blood glucose level was measured once more at the end of the operation. The patients who required more than 3 carpules of anesthetic agent were excluded from the study. This could be due to either feeling pain, or in the instances that the operation took longer than 90 minutes.

Data were analyzed by SPSS software Ver3 , using ANOVA test for repeated data, paired sample t-Test and Pearson correlation test. The P-value was adopted 0.05 and the confidence coefficient was assumed 95%.

## Results

17 (51%) patients were selected for the midazolam and 16(49%) in the propofol group. There were no significant statistical differences between the groups regarding the number of carpules, participants’ average age, surgery duration and the pre-operational DAS stress score (Table 1).

**Table 1:** The average age, duration of the dental implant surgery , number of injected carpules, pre-operational DAS stress score in both midazolam and propofol group.

**Group**	**Average age**	**Surgery duration**	**Number of injected** **carpules**	**Pre-operational DAS stress score**
Midazolam	45.76	51.67	2.29	11.58
Propofol	44.06	55.93	2.43	10.50
P-value	0.743	0.327	0.370	0.241

**Figure 1:**
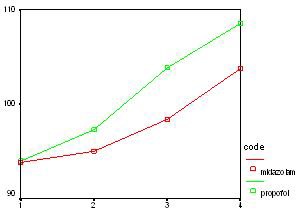
Average blood glucose level in different stages of the study

The average blood glucose level in the midazolam group before the operation was 93.82 mg/dl which revealed an increase during the operation and finally reached 103.76 mg/dl. The propofol group had an average blood glucose level of 94 mg/dl which increased to 108.56 mg/dl at the end of the operation (Figure 1 and Table 2).

**Table 2:** Average blood glucose level in different stages of study.

**Group**	**Average blood glucose level before operation**	**Average blood glucose level 2 minutes after local anesthetic injection**	**Average blood glucose level 30 minutes after beginning of operation**	**Average blood glucose level at the end of operation**	***p *** **Value**
Midazolam	93.82±14.71	95.00±12.93	98.41±12.64	103.76±15.08	*p* < 0.001
Propofol	94.00±13.22	97.31±12.65	103.81±12.15	108.56±9.95	*p* < 0.001

The ANOVA test for repeated data between the average blood glucose levels in different stages and between the 2 groups was performed and the results are depicted in Table 1. There was no statistically significant difference between the 2 groups during different stages of the current study (*p*= 0.0466). In both, the midazolam and the propofol groups, the average blood glucose had a statistically significant increase in all 4 stages (Table 2).

The relation between pre-operation DAS stress score and the average blood glucose level in all 4 stages, using the Pearson correlation coefficient (Table 3) showed statistically meaningful relation between the pre-operation DAS stress score and the average blood glucose level in all 4 stages of study in midazolam group (*p*< 0.001). A statistically significant increase in the average blood glucose level in all stages was found as the pre-operation DAS stress scores increased (r= 0.832, *p*< 0.001).The analysis also revealed that the pre-operation DAS stress score and the average blood glucose level in the propofol group, before the operation and 2 minutes after the injection of anesthetic, had a statistically significant direct relation. The average blood glucose level raised when the pre- operational DAS stress score increased (*p*< 0.024, r= 0.035).

**Table 3:** The frequency distribution of the average blood glucose level in both groups regarding the stress score

**Stress score**	**DAS**	*N	**Pre-operation average blood glucose level**		**Average blood glucose level 2 minutes after anesthetic injection**		**Average blood glucose level 10 minutes after beginning of operation**		**Average blood glucose level at the end of operation**	
No stress	4-8	4	89	95.5	91.5	98	96	104	99.5	109.5
Moderate stress	9-12	19	83.7	90.7	86	92.9	89.2	100.4	93.2	105.5
Severe stress	13-14	7	105	103.2	107	108	111	112.2	117.6	115.7
Phobia	>15	3	115.6	-	112.3	-	115	-	124.3	-

N*: number of people

There was no statistically meaningful relation between the DAS stress score and the average blood glucose level of the propofol group, both measured 30 minutes after the commencement and the end of operation (*p*= 0.13, r= 0.052).

## Discussion

The blood glucose level changes during the dental surgeries [1-3]. Different factors such as diabetes, stress, trauma from surgery, injection of epinephrine-containing anesthetics and the medications used during surgery can alter the blood glucose level. It is difficult to dominate all these factors simultaneously; however, changes in the blood glucose level can be controlled by managing the stress [1-3, 5-8].

Currently, effortless measurement of the blood glucose level can be performed by using relevant glucometer devices during a dental implant surgery. Studies confirmed the precision of these devices to be similar to laboratory tests when total venous blood sample was used [19-20]. These devices are user friendly and by employing a non-aggressive procedure, they can provide rapid results [19-20].

The findings of the current study were almost in line with the results yielded by the study of Oztekin et al. performed in 2006 [16]. They studied the effect of midazolam and propofol on blood glucose level, blood lipid level and plasma osmolality in 5 steps during coronary artery bypass graft surgery in two groups, each including 10 patients [16]. They found that the blood glucose level has increased in both examined groups, although the difference between two groups was reported to be not statistically meaningful [16].

Myles et al. studied the changes in the serum glucose and blood glucose levels induced by propofol prescription during cardiac surgeries in 22 patients, in 10 different stages. They concluded that blood glucose has increased in different stages and this difference was statistically meaningful [21]. In our study, likewise, the sedated patients with propofol, showed a statistical significant increase in the blood sugar level.

In another study performed by Haghighat and Kaviani, the changes in the blood glucose level during extraction of impacted third molars, using an epinephrine-containing local anesthetic, were studied. In their study, no sedatives were prescribed and the blood glucose level was measured in 4 stages. It was concluded that the raise in the blood glucose level was statistically meaningful [22].

In our study, the level of the blood glucose was in direct relation with the DAS score.The difference between this study and the current study was the medication used in the experiment.

The findings of this study showed a meaningful relation between stress and the average level of blood glucose in different stages of measurement in both groups. As the pre-operation DAS stress score increased, the blood glucose level in both midazolam and propofol groups raised in direct relation. Stress can induce an increase in the blood glucose level and is considered as a physiologic mechanism. It seems that the increase in the blood glucose level during a surgery occurs generally, in patients who intake sedatives [16-17]. The operational and pre-operational stress has an imperative impact on the blood glucose by increasing its level during a surgical operation like a dental implant surgery. The sedation may reduce the extent of blood glucose raise in an operation and the current study experienced no statistical difference between midazolam and propofol.

The current study did not include a control group, following the method employed by previously published studies [16-17].

## Conclusion

Based on the results yielded by this study; patients who receive venous sedation, either by midazolam or propofol, exhibit an increase in the blood glucose level when undergoing an operation. No statistically significant difference is detected between midazolam and propofol. The prescription of midazolam and propofol as the sedative medications in the dental implant surgery increases the blood glucose level during the operation which would be a crucial issue in high risk patients like diabetic patients.
